# The Ancestry of Chalicotherium

**Published:** 1892-06

**Authors:** Henry F. Osborn


### The Ancestry of Chalicotherium.

Chalicotherium is a genus which appears in the lower Miocene simultaneously in Europe and America, where it has been very recently discovered. It extends into the Pliocene and then disappears. It has attracted unusual attention of late, owing to the discovery by Filhol and independently by Forsyth Major that the foot-bones of *Macrotherium*, which has been considered an Edentate, really belong to *Chalicotherium*. As the teeth are wholly different from those of the Edentates, and similar to those of the Ungulates, this genus represents a very aberrant and unique family.

The only known Ungulates which present a dentition at all similar are *Palæosyops* and *Meniscotherium*. The latter is from near the base of the Eocene, and last year in analyzing its dentition I found so many very striking resemblances to that of *Chalicotherium* that I was led to suggest that *Meniscotherium* might be the long-sought ancestral form, reserving final judgment until the feet were discovered. Marsh has very recently figured the feet of *Meniscotherium* (*Hyracops*), and upon the whole, I think they sustain the supposition that the *Chalicotheriidæ* were derived from the *Meniscotheriidæ*. There are some profound differences, but these are mainly such as separate primitive from highly modified forms. The resemblances consist in the tridactylism of both genera and the marked similarity in tooth structure. I will discuss these points in more detail in the *American Naturalist* for June. HENRY F. OSBORN.

